# Thin Polymer Films by Oxidative or Reductive Electropolymerization and Their Application in Electrochromic Windows and Thin-Film Sensors

**DOI:** 10.3390/molecules28020883

**Published:** 2023-01-16

**Authors:** Ibeth Rendón-Enríquez, Alex Palma-Cando, Florian Körber, Felix Niebisch, Michael Forster, Michael W. Tausch, Ullrich Scherf

**Affiliations:** 1 Grupo de Investigación Aplicada en Materiales y Procesos (GIAMP), School of Chemical Sciences and Engineering, Yachay Tech University, Urcuquí 100119, Ecuador; 2 Department of Chemistry, Macromolecular Chemistry and Wuppertal Center for Smart Materials @ Systems (CM@S), Bergische Universität Wuppertal, Gaußstr. 20, 42119 Wuppertal, Germany; 3 Department of Chemistry, Chemical Education and Wuppertal Center for Smart Materials @ Systems (CM@S), Bergische Universität Wuppertal, Gaußstr. 20, 42119 Wuppertal, Germany

**Keywords:** electrically conducting polymers, electropolymerization, cyclovoltammetry, electrochromic windows, microporous polymer networks

## Abstract

Electrically conducting and semiconducting polymers represent a special and still very attractive class of functional chromophores, especially due to their unique optical and electronic properties and their broad device application potential. They are potentially suitable as materials for several applications of high future relevance, for example flexible photovoltaic modules, components of displays/screens and batteries, electrochromic windows, or photocatalysts. Therefore, their synthesis and structure elucidation are still intensely investigated. This article will demonstrate the very fruitful interplay of current electropolymerization research and its exploitation for science education issues. Experiments involving the synthesis of conducting polymers and their assembly into functional devices can be used to teach basic chemical and physical principles as well as to motivate students for an innovative and interdisciplinary field of chemistry.

## 1. Introduction

**Figure 1:**
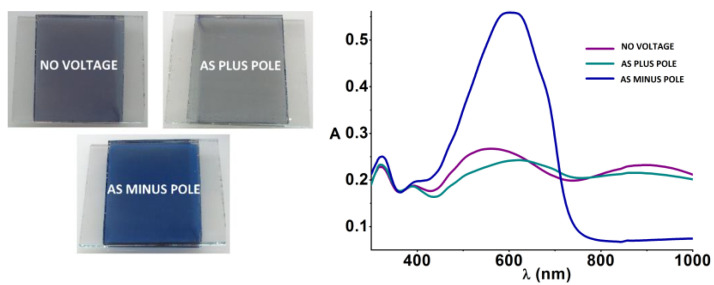
Colours of the electrochromic window made of PEDOT/PDTBT without and with applied voltage (forward/reversed bias), and the corresponding transmission spectra [2].

**Scheme 1:**
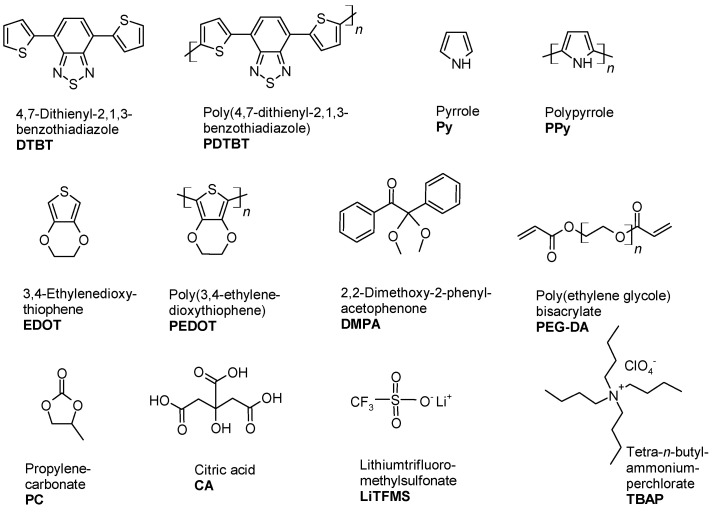
Monomers, polymers and additives used for the synthesis of the electrochromic windows.

The cooperation between different branches of chemical science, especially involving groups that are engaged in chemical education, very often depends on the presence of common touchpoints. This joint report wants to document the challenging and very fruitful interaction of two research groups, working in the different areas of chemical education and polymer science, respectively, within the common topic “electropolymerization”. This work was driven by jointly supervised PhD students as well as joint external funding, resulting in remarkable knowledge gain and with mutual benefit for both parties. Let us begin our short journey with linear (semi)conducting and electroactive polymers that are generated in oxidative electropolymerization schemes and their science education implications, especially concerning their electrochromic properties. For use in educational experiments, we have recently developed, assembled, and described an easily implementable electrochromic window as shown in Figure 1. This simple demonstrator is based on electrochromic devices based on poly(3,4-ethylenedioxythiophene) PEDOT as active material, as known from the literature [1] and as adopted by us in 2020 [2]. This assembly process is characterized by the following features: (i) The electroactive devices include two (semi)conducting polymers, PEDOT and poly(4,7-dithienyl-2,1,3-benzothiadiazole) PDTBT, in their active layer (see Scheme 1). (ii) The devices are built as a model of a dry window with double glazing, without fluid components. (iii) The assembly process uses two different types of polymerization: electrochemical polymerization for fabrication of the (semi)conducting polymers as well as photochemical initiated radical polymerization for the generation of a suited solid electrolyte layer, and (iv): The needed electrical/ion conductivity is ensured by ionic dopants, that are mixed and embedded into the cross-linked poly(ethylene glycol) diacrylate PDA (see Scheme 1) solid-state electrolyte interlayer.

**Figure 2:**
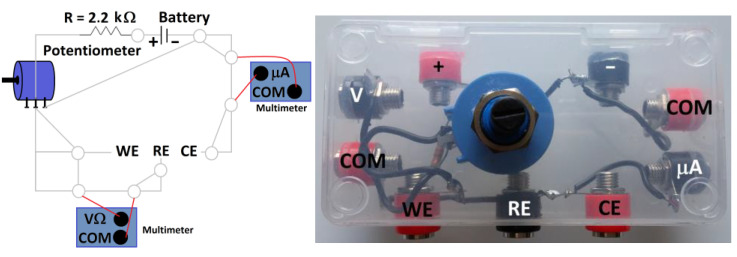
Circuit diagram and photographic image of the potentiostat used for our electrochemical depositions.

**Figure 3:**
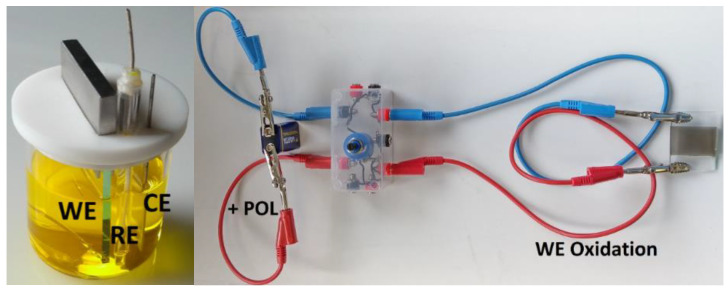
Synthesis cell for electrochemical polymerizations and equipment for measuring the device parameters of the electrochromic windows with double glazing.

Equipment for the preparation and investigation of electrochromic windows: The two main components of the equipment for the preparation and investigation of electrochromic windows with (semi)conducting polymers as active materials are a potentiostat and an electrosynthesis cell. The simplified devices shown in Figure 2 and Figure 3 were designed, developed, and tested in cooperation with José Alejandro Baeza Reyes (UNAM Mexico City, Mexico).

The potentiostat ensures a precise control and adjustment of the electrical parameters both during electrochemical polymer synthesis and in the characterization of the electrochromic properties of our electrochromic windows.

The equipment also includes an UV lamp (laboratory hand lamp) or UV LEDs (λ = 365 nm) to drive the photopolymerization and an UV-VIS spectrophotometer to record the solid-state transmission (absorption) spectra. Detailed instructions for the assembly of the potentiostat and the synthesis cell as well as for the investigation of the electrochromic windows are available online (Ph.D. thesis of I. N. R.-E.) at https://chemiemitlicht.uni-wuppertal.de/de/dissertationen.html [3]. Scheme 1 shows the chemical formula, names and abbreviations of the main compounds used in the synthesis of the layers as well as the assembly and characterization of the electrochromic properties of the double-glazed electrochromic window from Figure 1. The selection includes: (i) Three monomers for the synthesis of the corresponding (semi)conducting electrochromic polymers (PDTBT, PEDOT and PPy), (ii) the prepolymer PRG-DA and the radical initiator DMPA for photopolymerization of the solid-state electrolyte layer that connects the two polymer-coated glass substrates, and (iii) additives (Li-TFMS, TBAP) that provide sufficient charge carrier mobility during electropolymerization (TBAP) and in the electrochromic device (LiTFMS).

## 2. Methods of Electropolymerization

**Figure 4:**
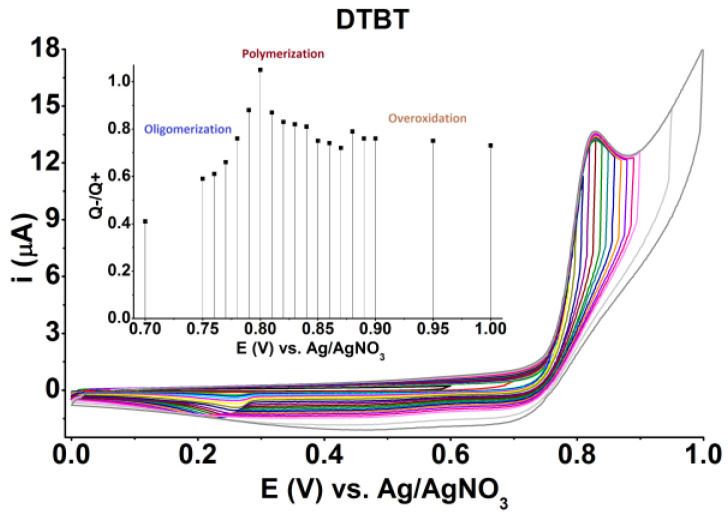
Orienting cyclovoltammetric measurements to determine the optimal working voltage for the electrochemical, oxidative deposition of monomer DTBT.

Before discussing the assembly of our electrochromic windows, let us briefly outline some general features of electropolymerizations. When tackling the electropolymerization of a new electroactive monomer, it is first appropriate to determine the optimum voltage at which the planned (electro)polymerization will be carried out. The three monomers DTBT, Py, and PEDOT, depicted in Scheme 1, (and others, e.g., bithiophene BT, carbazole Cz and aniline Ani) all polymerize oxidatively at the positive pole of the electrosynthesis cell, with cationic intermediates generated by anodic oxidation. If the voltage is too low, polymerisation does not occur at all or occurs only under formation of short chain oligomers. If the voltage is too high, so-called “over-oxidation” is the consequence, connected to the formation of structural defects. As an example, Figure 4 depicts the cyclovoltammetric (CV) current-voltage curves when electropolymerizing the dithienylbenzothiadiazole monomer DTBT into the corresponding polymer PDTBT (see Scheme 1). The differently coloured plots represent the current-voltage curves obtained from voltage cycles under repeated changes of the peak voltage at the working electrode, always starting at 0 V vs. Ag/AgNO_3_ and stepwise increasing the voltage to 0.7–1.0 V. In the inset diagram, the ratio Q-/Q+ represents the sum of the negative and positive charge carriers that are accumulated in one cycle. The ratio Q-/Q+ is calculated as the integral ratio between the respective current curves and the axis corresponding to a current of i = 0 A. In the DTBT case, the evaluation of the CV plots documents that the polymerisation proceeds most efficiently at a peak voltage of 0.8 V vs. Ag/AgNO_3_ [4,5].

Generally, electropolymerized films can be prepared by anodic oxidation or cathodic reduction from the respective monomers, with a predominant emphasis on anodic, oxidative processes in the literature until today. In Chapter 5, we will also give one example for until now underexplored cathodic, reductive processes. In any case, the nanoscopic structure, in particular the homogeneity, roughness, and porosity, as well as the adhesion of the deposited polymer layer to the electrode surface must be well controlled (see Figures 8 and 9 discussed later). From a mechanistic point of view, when the oxidative (or reductive) voltage is applied, a Faraday current develops due to an oxidation or reduction of the electroactive monomer. Hereby, a concentration gradient is generated in the vicinity of the electrode surface, connected to the formation of a so-called “diffusion layer” in which a concentration balance between the solution phase and the electrode surface occurs. The thickness of the diffusion layer increases with an increase in the applied potential.

**Figure 5:**
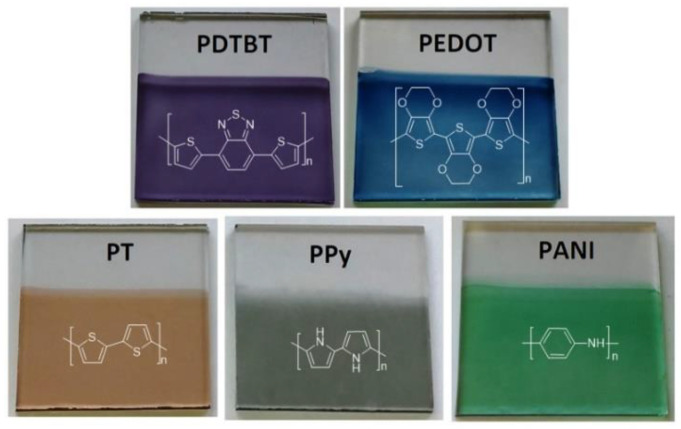
Polymer layers on FTO glass substrates obtained by oxidative electropolymerization.

The well-characterized oxidative electrochemical polymerizations consist of several reaction steps in which radical cations and oligomeric dictations are involved as intermediates [6,7,8,9,10,11,12]. Since the required potential E° for the oxidation of the monomer is always higher than that for the charging of the oligomeric intermediates and the resulting polymer, many processes take place simultaneously, including chain formation and doping/dedoping processes, finally resulting in the formation of the desired polymer. With the help of such oxidative polymerizations, electrochromic layers can be produced on top of FTO glass substrates in a few minutes at deposition voltages of 0.6–1 V (vs. Ag/AgNO_3_, Table 1, Figure 5).

## 3. Assembly and Operation of Electrochromic Windows

Let us switch back to the generation of our electrochromic windows. Two FTO glasses, one of them already electrochemically coated with a thin layer of polymer PDTBT (see Scheme 1), are assembled together with the help of a gel-like mixture that contains (i) the photochemically curable poly(ethylene glycol) acrylate PEG-DA precursor, (ii) the photoinitiator 2,2-dimethoxy-2-phenylacetophenone DMPA, (iii) the electrochemically polymerizable EDOT monomer, and (iv) LiTFMS as ion-conducting additive that enables the electrical conduction between the two FTO glasses. As a result, LiTFMS is incorporated/dispersed into the crosslinked polymer layer. In terms of processing, the uncoated FTO glass is coated with a gel layer consisting of EDOT, DA, DMPA and LITFMS. Next, EDOT is electrochemically polymerized to PEDOT with a DC voltage source. Then, the second FTO glass is placed on top and the final photochemical crosslinking of the PEG-DA/DMPA system under formation of the solid electrolyte layer is initiated with a laboratory hand lamp (λ = 365 nm) (see [2] for a detailed procedure).

For checking the electrochromic characteristics of the assembled electrochromic window, the two RE/GE sockets at the potentiostat are shorted via a cable. A 9 V battery is connected to the potentiostat as indicated in Figure 3. A positive voltage at one polymer-coated glass leads to oxidation (oxidative doping) of the polymer material, a negative voltage to reduction (reductive dedoping). To change the polarity, the positive pole and negative pole can be exchanged.

**Figure 6:**
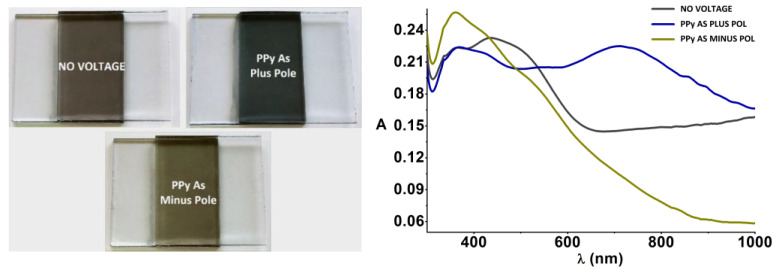
Electrochromic colors of polypyrrole (PPy)-based electrochromic windows, and the corresponding transmission spectra.

As a model system with only one (semi)conducting polymer, a polypyrrole PPy-based electrochromic window in which the second FTO glass is not coated with a second (semi)conducting polymer shows a grey to black color depending on the thickness of the PPy layer and the doping level. When PPy is fully reduced to the neutral state (connection to the negative -minus- pole), the color changes to yellowish (see Figure 6). When the PPy is oxidized (connection to the positive -plus- pole), a blueish color appears. The absorption spectrum now shows a long wavelength, doping-induced maximum at approx. 770 nm (Figure 6).

Electrochromic windows with polythiophene (PT) and polyaniline (PAni) as active materials basically behave similar, of course with changes of the observed colors. In the following we will present some general considerations to explain colors and transmission/absorption spectra of the PPy-based electrochromic window of Figure 6: We assume that delocalized π-electron orbitals are extended over chain segments of specific length (so-called effectively conjugated segments). The energetic positions of these orbitals can be handled like valence band VB and conduction band LB in an inorganic semiconductor. The energy difference between the upper edge of the VB (corresponding to the HOMO) and the lower edge of the CB (corresponding to the LUMO), the so-called band gap, is defined by the chemical structure of the semiconducting polymer, here PPy, and the electronic and steric boundary conditions for the formation of these delocalized molecular orbitals. This band gap energy, and the resulting electronic absorption spectrum, are strongly influenced by so-called doping/dedoping processes, as already mentioned [13].

**Figure 7:**
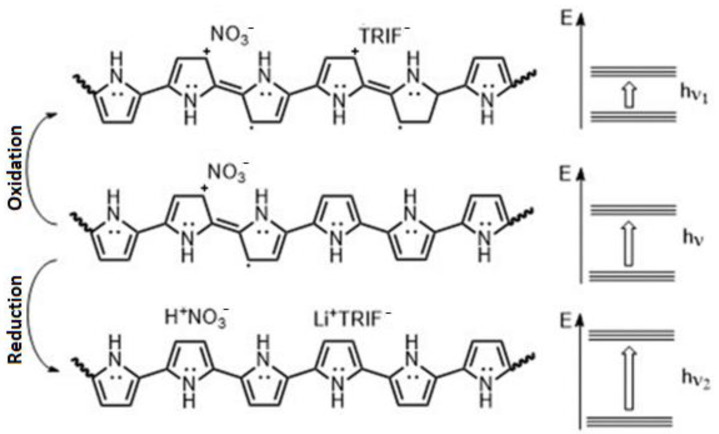
Doping and dedoping of polypyrrole PPy layers and didactic model for the changes of the color of the layers.

Figure 7 illustrates this doping/dedoping processes with (idealized) chemical structures of PPy chain segments, together with schematic energy levels of the absorbed photons. (i) Anodic polymerization initially results in the presence of oxidatively formed, radical-cationic polymer segments, called polarons. Such a structural segment is depicted in the middle part of Figure 7 and represents a partially doped (oxidized) state. (ii) During further doping by anodic oxidation at the positive pole, more radical-cationic units are generated (and possibly also double positively charged segments as so-called bipolarons, that are not depicted here), resulting in a reduced band gap energy (Figure 7, top). Lower-energy photons are now absorbed and the color of the layer is shifted towards black. (iii) Dedoping by reduction at the minus pole leads to a removal of radical-cationic polarons (Figure 7, bottom). In the corresponding chain also the planarity of neighboring heteroaromatic units is reduced, leading to a more distorted structure and a reduction of the π-electron delocalization. As a result, the gap between valence and conduction band increases, and the visible color of the PPy layer shifts towards yellow, that means towards higher energy.

In the case of our electrochromic windows with two FTO glasses that are coated with two different electroactive polymers, e.g., in the device of Figure 1, the absorption spectra and thus the resulting colors of the windows are additive, that means are a superposition of the existing redox states of both polymers, and are influenced by layer thickness and film morphology [14,15,16]. Here, a clear relation of structural elements (occurrence of polarons and bipolarons) to the observed color of the window is much more complex, since the active layer containing two electroactive polymers can exist at several, different doping states for a given potential/voltage.

## 4. Adhesion of Electrochemically Deposited Films and Thin Film Morphology

**Figure 8:**
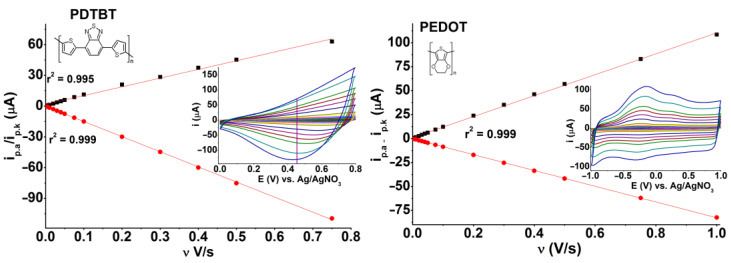
Plots of the reductive and oxidative currents maxima against the potential feed rates (scan rates) for films of electrochemically synthesized polymers PDTBT and PEDOT (the chemical structures of the polymers are shown as insets).

For all discussed redox properties in our devices, assembled as described above, a sufficient electrical contact of the formed polymer layers with the electrode surface is crucial (i.e., the sufficient adhesion of the films to the electrode surface, at least to an extent that an effective charge transport through the as-formed layers is achieved). To prove this prerequisite cyclic voltammograms are recorded at different feed rates of the electrode potential (“scan rates”) and plotted against the current at a certain potential (usually in the peak maxima) (Figure 8). The observed linear dependence between scan rate and peak current demonstrates sufficient adhesion of the polymer films on the electrodes [17].

It should be noted, however, that in addition to a sufficient adhesion, all layers must also guarantee an effective charge transport in the solid (“bulk”) state, i.e., they must be at least semiconducting. This is usually the case for polypyrrole, polythiophene, or polyaniline. However, for other electropolymerization products (e.g., the later described polymer networks from reductive couplings of trihalogenated pyridyl monomers) problems with the charge transport through the deposited layer can occur for an increased layer thickness of the polymer films, as documented by decreased peak currents.

**Figure 9:**
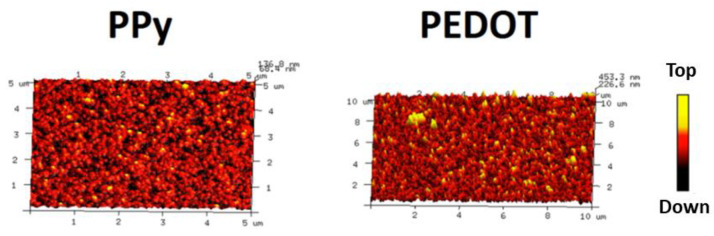
Three-dimensional AFM (Atomic Force Microscopy) images of representative sectors of the PPy and PEDOT surfaces.

Electropolymerized layers generally show a typical, granular morphology, caused by the localized and irregularly starting nucleation process on the electrode. Although, the AFM images from Figure 9 show that the electrochemically synthesized polymer layers exhibit certain morphological differences. For the cutout of the surface of a PPy layer in Figure 9, the difference between highest peak and deepest valley is approx. 68 nm, as a measure of the bumpiness of the layer. For the PEDOT layer this difference is, with approx. 227 nm, around three times higher. This translates into a mean average roughness of 14 nm for the PPy and 43 nm for the PEDOT layer.

## 5. Electrochemical Deposition of Polymer Networks

**Figure 10:**
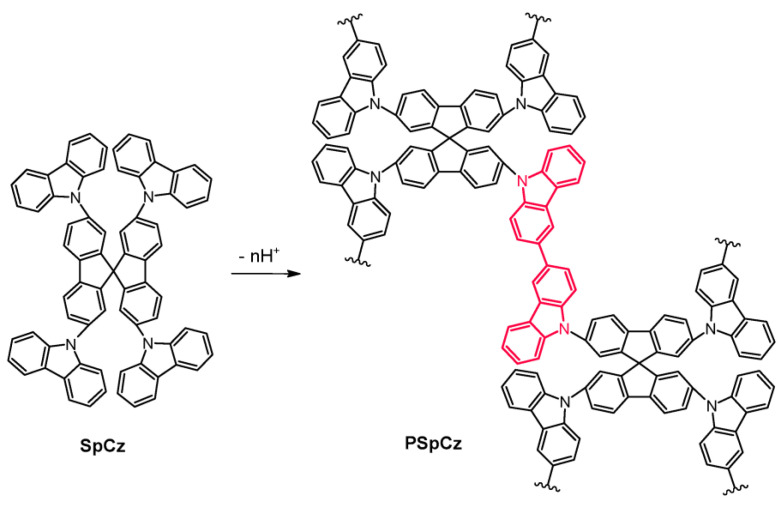
Oxidative electropolymerization of the tetrafunctional monomer SpCz into the corresponding polymer network PSpCz.

**Figure 11:**
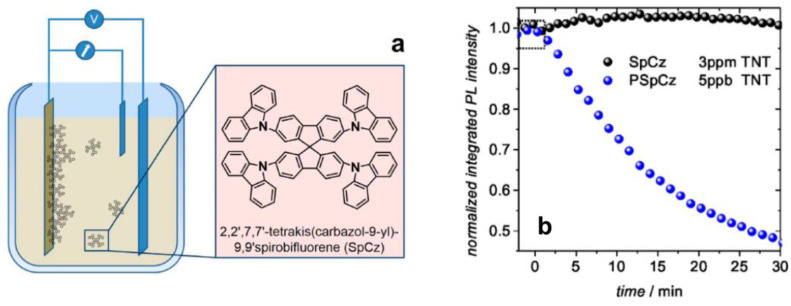
Application example of electrochemically deposited microporous polymer networks in thin film fluorescence sensors for nitroaromatic analytes: (**a**) generation of microporous PSpCz films-the inset shows the chemical structure of the monomer SpCz that have been used for electrochemical polymerization; (**b**) optically detected fluorescence quenching of PSpCz by TNT as analyte (PSpCz: electrochemically deposited microporous polymer film, SpCz: non-porous film of the vacuum-deposited monomer, prepared for comparison).

Another scientifically interesting feature of electropolymerizations, now by switching our interest to novel polymer materials made by electropolymerization, is the possibility of depositing thin films of insoluble polymer networks directly on top of the electrodes. These products can be deposited as homogeneous layers on the electrode surface by coupling rigid monomers of higher functionality. The deposits often show an additional, permanent microporosity. For that, in contrast to the synthesis of linear polymers from difunctional monomers, monomers containing more than two reactive functions are used (see Figure 10 for a tetrafunctional carbazole derivative SpCz with a spirobifluorene core segment) [18,19]. Such networks can be generated in an oxidative dimerization of carbazole functions as elementary, network-forming reaction [18,19]. The product PSpCz obtained in this procedure is deposited as thin film on the working electrode and can exhibit very high intrinsic surface areas (determined by the BET method with Krypton gas as sorbent) of >2000 m^2^/g, with average pore diameters of 1–2 nm. This intrinsic microporosity causes a significantly increased analyte sensitivity when applying such electropolymerized films as optical, thin-film fluorescence sensors for nitroaromatic analytes (in the turn-off-mode). For instance, in the case of 2,4,6-trinitrotoluene TNT as analyte, sensitivities up to the ppb detection range (5 ppb, Figure 11) have been observed and reported [20].

**Figure 12:**
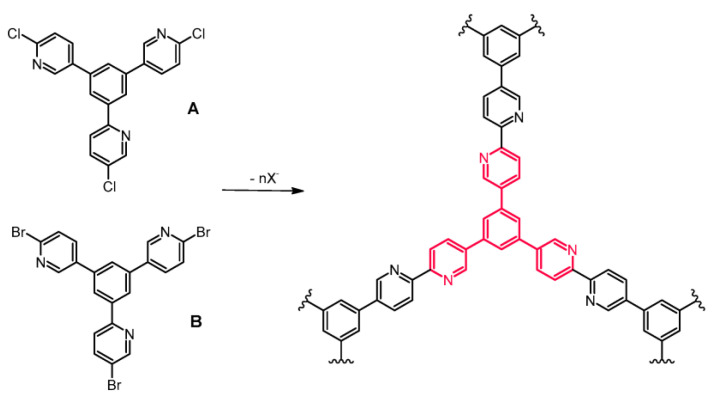
Reaction scheme of the reductive, electrochemical polymerization of the trifunctional monomers 1,3,5-tris(2-chloropyrid-5-yl)benzene **A** and 1,3,5-tris(2-bromopyrid-5-yl)benzene **B** (X: Cl or Br).

In general, and as already said, also reductive, electrochemical polymerizations are possible, but they are widely underexplored [21,22,23]. We would like to demonstrate that this principle can be also applied for the electrochemical synthesis of polymer networks, i.e., in a deposition of an insoluble film of the target polymer network directly on the working electrode. For this purpose, and as already demonstrated above for the oxidative electropolymerization, we use monomers that contain more than two reactive groups. In contrast to the elimination of protons in the course of oxidative couplings, now negatively charged fragments are eliminated, in our example halide anions. In such an electrochemical coupling of electron-deficient monomers, here trifunctional 2-halopyrid-5-yl derivatives with a 1,3,5-trisubstituted benzene unit as the core segment (see Figure 12), primarily anionic intermediates are generated by electron injection from the cathode as negative electrode. These intermediates then couple by halide anion elimination and dimerization [24].

**Figure 13:**
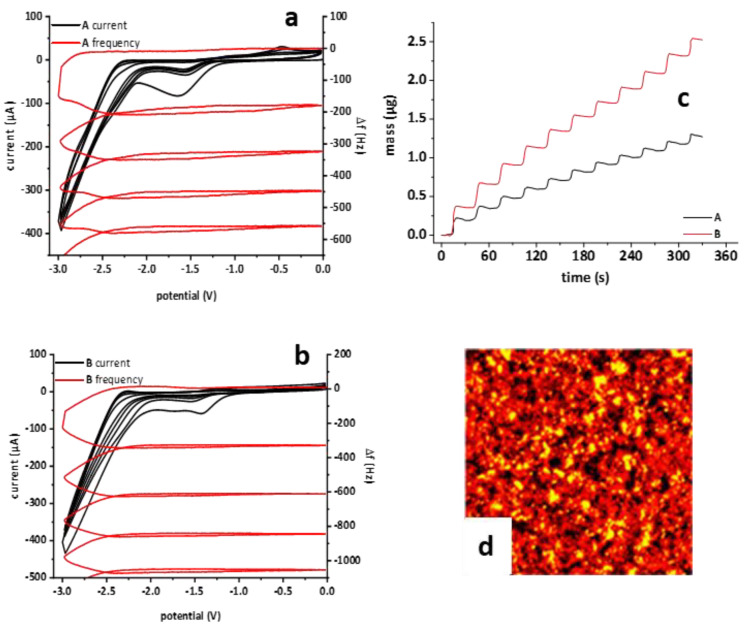
(**a**,**b**): Reductive, electrochemical polymerization of the trifunctional monomers 1,3,5-tris(2-chloropyrid-5-yl)benzene **A** and 1,3,5-tris(2-bromopyrid-5-yl)benzene **B**; cyclic voltammograms of electropolymerization and gravimetric tracking of film deposition by the electrochemical quartz crystal microbalance method (monomer concentration c = 5 mM, conducting electrolyte: 0.1 M TBABF4 in DMF, feed rate v = 0.1 V/s, working electrode: Pt disk 0.198 cm^2^, reference electrode: Ag/Ag^+^, Df is the frequency shift of the quartz crystal); (**c**): Calculated stepwise mass increase of electrodeposited polymer films starting from monomers **A** and **B**. The AFM image (**d**) shows the typical granular morphology of a polymer layer formed from monomer **A**, see Chapter 4 of this study, (edge length: 2.5 × 2.5 mm), from Ref. [24].

A suitable method for tracking the film deposition process are gravimetric measurements using the so-called electrochemical quartz crystal microbalance EQCM. Figure 12 and Figure 13 show the reductive, electrochemical deposition of polymer films (as polymer networks), starting from the monomers 1,3,5-tris(2-chloropyrid-5-yl)benzene or 1,3,5-tris(2-bromopyrid-5-yl)benzene on a platinum-coated quartz crystals (monomers **A** and **B**). The decrease in the oscillator frequency (Figure 13) of the quartz crystal (frequency f in Hz) reflects the mass increase of the deposit at the electrode and demonstrates the gradual, stepwise build-up of the growing polymer layers (with cyclic potential control in the potential range between 0 and −3 V) [24].

However, it must be noted that both oxidative and reductive electropolymerizations are not radical or ionic chain growth polymerization processes, because each subsequent step of polymer grow is initiated by a new electron transfer step to the anode or from the cathode, respectively. For this reason, and from the mechanistic point of view, the polymer-forming processes are polycondensations.

## 6. Materials and Methods

### 6.1. Reagents and Methods

All reagents and chemicals were purchased from commercial companies, unless otherwise stated. Acetonitrile (ACN) and dichloromethane (HPLC grade) were refluxed over calcium hydride and phosphorus pentoxide, respectively, and distilled. Tetrabutylammonium perchlorate (TBAP, for electrochemical analysis, ≥99.0%) was purchased from Sigma-Aldrich. ^1^H and ^13^C NMR spectra were obtained on Bruker Avance 400 and III 600 machines. APLI and ESI mass spectra were recorded on a Bruker Daltonik micrOTOF system (KrF*-Laser ATLEX-SI, ATL Wermelskirchen), MALDI-TOF mass spectra on a Bruker Reflex TOF. IR spectra were recorded on a JASCO FT/IR spectrometer with an ATR unit.

For electrochemical polymerization and characterization, an electrochemical workstation PAR VersaSTAT 4 was used in combination to a three-electrode cell and connected to a QCM922A oscillator for the EQCM experiments. The AFM images were obtained with an atomic force microscope (AFM) BrukerdiInnova operated in tapping mode. Average surface roughness and thickness of the films were extracted from the topography images. The electrochemical quartz crystal microbalance (EQCM) technique, also called electrogravimetry, represents a powerful method for recording small mass changes (up to the pg scale) at the electrodes. The method can be coupled with simultaneous electrochemical measurements, e.g., cyclic voltammetry. The mass variations result from frequency changes (f) of a quartz crystal resonator.

### 6.2. Determination of the Optimum Voltage for Electropolymerization

To determine the optimum voltage for electropolymerization, the electrochemical behaviour of the monomers was studied on a platinum disk electrode. The solution for the electropolymerizations contained monomer, solvent, and conducting salt as described in Table 1. First, argon was bubbled through the solution for 10 min. During the measurement, a constant argon stream was passed over the solution. For every voltage range only the first voltage cycle was analysed.

### 6.3. Assembly and Operation of Electrochromic Windows

The manufacture of the electrochromic windows is based on references [1,25,26]. However, the electropolymerization was carried out with a self-made potentiostat and a self-made electrosynthesis cell. The procedure involves the following steps:

(1) A FTO-glass slide covered with the respective electropolymerized layer was coated with two drops of an “adhesive” mixture. This mixture was prepared as 1:1 blend of PC and PEG-DA (see Scheme 1). The mixture also contains DMPA (0.02 mol/L) and LITFMS (0.1 mol/L, see Scheme 1). The “adhesive” mixture was prepared in a small brown glass container by mixing 1 mL PC, 1.3 g of PEG-DA, 5.0 mg of DMPA and 0.2 g of LITFMS. This mixture was homogenized in an ultrasonic bath for 15 min.
(2) A second untreated, but clean FTO-glass was placed on top. The “adhesive” mixture was homogeneously distributed between the two glass slides by squeezing.
(3) To accomplish the photo-crosslinking of PEG-DA, the loosely connected glass slides were irradiated with a UV-lamp at a λ = 365 nm for 15 min.

### 6.4. Adhesion of the Electrochemically Deposited Films

The adhesion of the films at the electrode surfaces was tested using cyclovoltammetric measurements under the conditions as described in Table 1, but without monomer. The scan rate was systematically varied, the cathodic and the anodic peak currents were recorded and plotted against the scan rates.

### 6.5. Oxidative Electrochemical Polymerization of SpCz and Characterization of PSpCz on ITO Electrodes

The electropolymerization was carried out in a three-electrode cell under argon atmosphere at 25 °C as described in [14]. ITO (ca. 1,8 cm^2^ deposition area) on glass and a platinum gauze (2.5 × 1.2 cm^2^ area), separated at a distance of 1 cm, were used as working (WE) and counter electrode (CE), respectively, together with Ag°/AgNO_3_ as reference electrode (RE). For morphology characterization and optical sensing experiments, films of the microporous **PSpCz** networks on ITO were prepared in a potentiostatic electropolymerization by applying a constant oxidative potential of 1.0 V for 20 min. Finally, a negative potential of -1.0 V was applied for 60 s for dedoping the deposits. After rinsing the films with acetonitrile and dichloromethane, the films were dried for 20 min at 100 °C. The resulting deposits on ITO were electrochemically characterized in 0.1 M (monomer-free) solutions of TBAP in acetonitrile.

### 6.6. Monomers for Reductive Electrochemical Polymerization

1,3,5-Tris(2-chloropyrid-5-yl)benzene (**A**).

Under an argon atmosphere 1,3,5-tris(4,4,5,5-tetramethyl-1,3,2-dioxaborolan-2-yl)benzene (1.433 g; 3.14 mmol), potassium carbonate (2.17 g; 15.72 mmol), 2-chloro-5-iodopyridine (2.84 g; 11.63 mmol), and Pd(PPh_3_)_4_ (0.225 g; 0.314 mmol) were suspended in 20 mL of dimethylformamide (DMF). The reaction mixture was heated up to 100 °C and stirred for 16 h at this temperature. Next the reaction mixture was stopped by addition of water. The precipitated solid was filtered off and washed, first with water and then with acetonitrile. Yield: 0.73 g (56%) of a white solid.

^1^H NMR (600 MHz, CDCl_3_) δ(ppm) = 8.68 (d, *J* = 2.6 Hz, 1H), 7.84 (dd, *J* = 8.2, 2.6 Hz, 1H), 7.74 (s, *J* = 5.9 Hz, 1H), 7.66 (d, *J* = 8.2 Hz, 1H). ^13^C NMR (151 MHz, CDCl_3_) δ(ppm) = 148.5, 142.0, 138.9, 137.1, 134.9, 128.3, 125.8. IR (neat): ṽ (cm^−1^) = 3047 (vw, C-H), 2976 (vw, C-H), 2921 (vw, C-H), 2857 (vw, C-H) 1602 (w, C=C), 1584 (w, C=C), 1559 (w, C=C). HR-MS (ESI): found: *m*/*z* = 412.0197 [M + H]^+^; calculated for C_21_H_13_Cl_3_N_3_: 412.0170.

1,3,5-Tri(2-bromopyrid-5-yl)benzene (**B**).

Under an argon atmosphere 1,3,5-tris(4,4,5,5-tetramethyl-1,3,2-dioxaborolan-2-yl)benzene (0.8 g; 1.75 mmol), potassium carbonate (1.21 g; 8.8 mmol), 2 bromo-5-iodopyridine (1.84 g; 6.5 mmol), and Pd(PPh_3_)_4_ (0.123 g; 0.175 mmol) were suspended in 20 mL of DMF. The reaction mixture was stirred for 16 h at 100 °C. The reaction was stopped by addition of water. The precipitated solid was filtered off and washed, first with water and then with acetonitrile. Yield: 0.48 g (50%) of a slightly yellow solid.

^1^H NMR (600 MHz, CDCl_3_) δ (ppm) = 8.68 (d, *J* = 2.6 Hz, 1H), 7.84 (dd, *J* = 8.2, 2.6 Hz, 1H), 7.74 (s, *J* = 5.9 Hz, 1H), 7.66 (d, *J* = 8.2 Hz, 1H). ^13^C NMR (151 MHz, CDCl_3_) δ (ppm) = 148.5, 142.0, 138.9, 137.1, 134.9, 128.3, 125.8. IR (neat): ṽ (cm^−1^) = 3051 (vw, C-H), 2922 (vw, C-H), 2852 (wv, C-H), 1598 (w, C=C), 1574 (w, C=C), 1552 (w, C=C). HR-MS (ESI): found: *m*/*z* = 543.8656 [M + H]^+^; calculated for C_12_H_13_Br_3_N_3_: 543.8654.

### 6.7. Reductive Electrochemical Polymerization of Monomers A and B on ITO or Pt/Quartz Crystal Electrodes (EQCM)

The reductive electrochemical polymerization of **A** and **B** was carried out in a three-electrode cell under argon atmosphere at 25 °C on ITO glass plates (12.5 mm × 40 mm, from PGO GmbH, ca. 1.8 cm^2^ deposition area) as working electrode. The counter electrode was a platinum-coated metal mesh (10 mm × 25 mm), with a cell volume of 10 mL. An Ag/Ag^+^ electrode with c(AgNO_3_) = 0.1 mol/L, c(TBAP) = 0.01 mol/L in acetonitrile (U = 0.60 V vs. NHE) was used as reference electrode (RE). For EQCM measurements a platinum disk on a quartz crystal with a = 0.198 cm^2^ has been used as working electrode (WE). Again, a platinum wire with d = 0.5 mm was used as counter electrode (CE) and an Ag/Ag^+^ electrode with c(AgNO_3_) = 0.1 mol/L, c(TBAP) = 0.01 mol/L in acetonitrile (U = 0.60 V vs. NHE) as the reference electrode (RE), with a cell volume of 20 mL.

*Electropolymerization on ITO*: 41.1 mg 1,3,5-tris(2-chloropyridin-5-yl)benzene (**A**, 10 mM) and 329.3 mg (0.1 M) TBABF_4_, or 54.6 mg 1,3,5-tris(2-bromopyridin-5-yl)benzene (**B**, 10 mM) and 329.3 mg (0.1 M) TBABF_4_ were dissolved in 10 mL of DMF in a three-electrode cell under argon atmosphere. Forty potential cycles from -3.0 V to 1.0 V were applied in the potentiodynamic mode with a scan rate of 0.2 V/s. The obtained films remain on the support (here the ITO plate). The films were washed with dichloromethane and dried.

IR (polymer film from **A**): ṽ (cm^−1^) = 1658 (w, C=C), 1598 (w, C=C). IR (polymer film from **B**): ṽ (cm^−1^) = 1655 (vw, C=C), 1598 (vw, C=C).

## 7. Outlook

Both oxidative and reductive electropolymerizations can be used to directly synthesize polymer films on the corresponding working electrode. These films can offer electrochromic, fluorescent, and in some cases intrinsically microporous properties. The functionality of the monomers that are used determines whether linear polymers or polymer networks are generated. Reductive electropolymerization is a still unexplored field; only a very few monomer types have been tested so far. Potentially suitable leaving groups for reductive electropolymerization are halide functions as well as aromatic diazonium or 4-cyanopyridinium groups, the latter remain to be investigated in detail.

In addition to the scientifically motivated development of the electrochemical fundamentals of oxidative and reductive electropolymerizations, applications of the produced thin films in electrochromic windows and optical sensors also play a key role in the cooperation project that is presented here, specifically in the demonstration of innovative applications of polymers in science education outreaches. The electrochromic windows based on polymer-coated FTO glasses are not only interesting for their electrochromic properties, such polymer-coated FTO glasses are also attractive for potential applications in photogalvanic or photovoltaic cells. In principle, they can occupy a central function in the conversion of light into electrical energy.

## Figures and Tables

**Table 1:** Polymer layers obtained by oxidative electropolymerization on FTO glass substrates (ACN: acetonitrile; aqueous buffer solution: pH 3, citric acid 0.8 mol/L and disodium hydrogen phosphate 0.05 mol/L).

Monomer	Concen-Tration (mmol/L)	Solvent	Conducting Salt Concentration (mol/L)	Deposition Voltage	Time (min)
DTBT	0.5	ACN	TBAP 0.1	0.8 V vs. Ag/AgNO_3_	2
EDOT	0.3	ACN	TBAP 0.1	1.0 V vs. Ag/AgNO_3_	5
Pyrrole	50	aqueous buffer solution	NaNO_3_ 0.1 HNO_3_ 0.1	0.6 V vs. Ag/AgCl	5
Aniline	400	water	H_2_SO_4_ 0.5	0.7 V vs. Ag/AgCl	5
Bithiophene	10	ACN	TBAP 0.1	0.85 V vs. Ag/AgNO_3_	5
DTBT EDOT	0.5 0.3	ACN	TBAP 0.1	0.8 V vs. Ag/AgNO_3_	5

## Data Availability

Not applicable.

## Sample Availability

Samples of the compounds are not available from the authors.
